# Perceptions of vulnerability to a future outbreak: a study of horse managers affected by the first Australian equine influenza outbreak

**DOI:** 10.1186/1746-6148-9-152

**Published:** 2013-07-31

**Authors:** Kathrin Schemann, Simon M Firestone, Melanie R Taylor, Jenny-Ann LML Toribio, Michael P Ward, Navneet K Dhand

**Affiliations:** 1 Faculty of Veterinary Science, The University of Sydney, Camden, NSW 2570, Australia; 2 Faculty of Veterinary Science, The University of Melbourne, Parkville, VIC 3010, Australia; 3 School of Medicine, University of Western Sydney, Penrith, NSW 2571, Australia

**Keywords:** Equine influenza, Perceived vulnerability, Protection motivation theory, Infection control, Biosecurity, Emergency animal disease

## Abstract

**Background:**

A growing body of work shows the benefits of applying social cognitive behavioural theory to investigate infection control and biosecurity practices. Protection motivation theory has been used to predict protective health behaviours. The theory outlines that a perception of a lack of vulnerability to a disease contributes to a reduced threat appraisal, which results in poorer motivation, and is linked to poorer compliance with advised health protective behaviours. This study, conducted following the first-ever outbreak of equine influenza in Australia in 2007, identified factors associated with horse managers’ perceived vulnerability to a future equine influenza outbreak.

**Results:**

Of the 200 respondents, 31.9% perceived themselves to be very vulnerable, 36.6% vulnerable and 31.4% not vulnerable to a future outbreak of equine influenza. Multivariable logistic regression modelling revealed that managers involved in horse racing and those on rural horse premises perceived themselves to have low levels of vulnerability. Managers of horse premises that experienced infection in their horses in 2007 and those seeking infection control information from specific sources reported increased levels of perceived vulnerability to a future outbreak.

**Conclusion:**

Different groups across the horse industry perceived differing levels of vulnerability to a future outbreak. Increased vulnerability contributes to favourable infection control behaviour and hence these findings are important for understanding uptake of recommended infection control measures. Future biosecurity communication strategies should be delivered through information sources suitable for the horse racing and rural sectors.

## Background

Improving the biosecurity practices of horse owners has recently become more important to animal health authorities due to increased spread of infectious diseases because of globalisation and more frequent animal movements. Many equine disease threats are enzootic, including leptospirosis and salmonellosis. Most previous work on zoonotic infection risks has been conducted with equine veterinarians, but one study also found animal handlers to be 10 times more likely than the public to have been exposed to *Coxiella burnetti*, the infectious agent of Q fever [1]. Salmonellosis and methicillin-resistant *Staphylococcus aureus* have been found to be responsible for disease outbreaks in equine clinical settings [2-4], and a similar trend of increased infection risk can be assumed for the acquisition of these infections by the horse owning public. Horse owners may also be more at risk than other members of the public to acquire other zoonotic infections such as other enteric bacteria and parasites or ringworm organisms. In Australia, zoonotic disease transmission from horses to humans has also caused significant health impacts and even human deaths in recent decades [5-7].

The potential of influenza viruses to infect mammalian hosts of multiple species and the recent crossover of equine influenza from horses to dogs [8], is worrisome with regard to future virus mutations. The 2007 equine influenza outbreak presents a unique opportunity to test a cognitive health behaviour theory empirically in relation to an animal disease outbreak setting. Application of the theory allows important lessons to be learnt for the delivery of health messages to the horse-owning public.

### Australia’s 2007 Equine influenza outbreak

Australia is one of the only three countries with substantial horse industries free of equine influenza, a highly contagious viral respiratory disease of horses, donkeys, zebras and mules [9]. In August 2007 Australia experienced its first ever outbreak of equine influenza. The outbreak lasted five months but during this time it affected more than 70 000 horses, spread over 280 000 square kilometres, and cost the government more than A$350 million before it was successfully controlled and the virus eradicated [10]. The full social and economic impacts of the outbreak are difficult to quantify [11], yet a study conducted during the epidemic found certain groups of horse owners to have much greater psychological distress compared to the general population [12]. Control strategies applied in the outbreak response included horse movement restrictions, emergency vaccination and enhanced on-farm infection control (biosecurity) and public awareness [13]. Horse movement restrictions were implemented immediately on detection of the outbreak and involved the cancellation of all horse gatherings and, initially, a nationwide ban on all horse movements. After 72 hours, a system of risk-based movement zones was established (involving restricted and controlled areas) in the two affected states (New South Wales (NSW) and Queensland) [14]. One month into the outbreak, at the commencement of the thoroughbred horse breeding season, a special restricted area was established, allowing horse movements within specified areas of high horse density [14]. Infection was already spreading rapidly in these areas and the cost to the horse industry was considered greater than the benefit of resource-intensive, local disease control efforts that were deemed unlikely to succeed [14,15]. Surrounding the special restricted area was a restricted area in which only very limited horse movements were allowed [14].

Despite the low mortality associated with disease and its ultimate eradication, horse owners and industry participants were greatly inconvenienced and suffered considerable financial and emotional hardships [12,16-19]. Commencing in early spring, the outbreak and associated movement restrictions, impeded horse travel for sporting and breeding purposes, imminent at that time of year. The mare is a seasonal spring breeder and spring is also associated with major horse racing carnivals and equestrian sporting events. In 2007, this included qualification events for the 2008 Beijing Olympic Games [10]. People deriving their primary income through horses or horse-related activities for example commercial stud farms, training centres, riding schools, veterinarians, farriers, horse chiropractors and dentists and horse event transport and catering service providers, all suffered economic impacts [17,19].

### Infection control behaviours (biosecurity compliance) amongst animal managers

**Figure 1:**
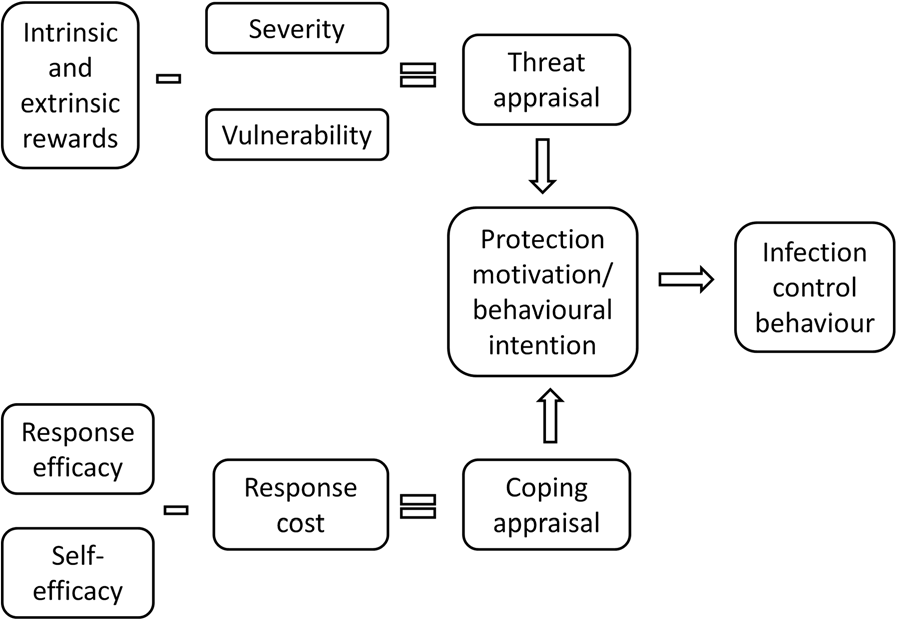
**Protection motivation theory as it applies to horse managers’ biosecurity behaviour (adapted from Rogers & Prentice-Dunn, 1975).**

The health of animals is directly influenced by the husbandry and infection control practices of their owners and handlers. Animal handlers’ actions however are an outcome of beliefs, perceptions and attitudes about those activities [20]. Several previous studies have linked the perceptions and attitudes of animal handlers to their infection control/biosecurity behaviour [20-24]. It is important to consider animal managers’ biosecurity risk perceptions and integrate them into animal health education campaigns in order for the campaign to be effective [20]. Much effort has been directed at developing animal disease management and eradication plans, including extension activities to raise the level of awareness amongst Australia’s horse industry [25]. In order to promote voluntary compliance of animal owners and managers, biosecurity and infection control programs need to be based on sound social cognitive models of protective health behaviour. One such model, previously applied to biosecurity behaviours of horse owners [26], is the protection motivation theory (Figure 1). The theory outlines two cognitive mediating processes forming a threat and a coping appraisal, respectively; these two processes combine to form protection motivation or an intention to perform a protective behaviour. The threat appraisal results from intrinsic or extrinsic rewards, such as social acceptance associated with not performing the behaviour, being counteracted by the perceived severity of the disease and perceived vulnerability. The coping appraisal focuses on actions an individual can take to protect his or her animals: this depends on an individual’s belief that a certain action or behaviour will reduce the threat (‘response efficacy’) and that they consider themselves able to carry out the protective measure (‘self-efficacy’). However, a number of response costs − such as availability of time, money or other resources to carry out the protective behaviour − may inhibit performance of the behaviour.

### Perceptions of vulnerability

According to the protection motivation theory, greater perceived vulnerability or susceptibility contributes to a threat appraisal, which, if raised in conjunction with adequate coping appraisal, influences behavioural intention and action positively [27,28]. However, some studies have found a negative relationship between perceived vulnerability and behavioural intention and action, which was attributed to denial or avoidance in response to great levels of anxiety in the absence of a sufficient coping appraisal [27]. These findings suggest that, generally a lack of perceived vulnerability is unfavourable to the motivation to perform a protective behaviour, whilst some degree of vulnerability acts to promote these behaviours up to a point where coping appraisal is insufficient.

Previous work suggests that perceived fear of a future equine influenza outbreak is significantly associated with implementing infection avoiding biosecurity practices [26]. Therefore, an understanding of horse managers’ perceived vulnerability and factors associated with those perceptions are important when attempting to develop tailored biosecurity education and extension campaigns.

This study identifies factors associated with levels of perceived vulnerability of horse owners/managers towards a future outbreak of equine influenza. The findings will help to explain how the experience of a disease outbreak influences perceptions of vulnerability to future outbreaks, and how this perception relates to preparedness. It will enable industry and government to improve the targeted delivery of biosecurity advice during future epidemics by considering the impacts of previous experience on the future engagement of the various segments of the target audience.

## Methods

### Questionnaire design, sampling and data handling

In this study, 270 premises were randomly selected from a dataset of premises supplied by the NSW Department of Primary Industries (DPI). To be eligible the premises had to be located in the restricted and special restricted areas of NSW during the 2007 outbreak, as designated according to the risk-based zoning system (described in detail in NSW DPI’s Equine Influenza Protection Plan [29]). Horse owners and managers of these premises (just called ‘managers’ hereafter) were enrolled via a personally addressed letter and up to three follow-up telephone calls. Of the 270 premises selected, 38 were deemed ineligible for various reasons (described elsewhere, [15]) whilst the managers of another 32 premises declined to participate. Further details on study design are presented in other published work, which analysed collected data separately to investigate factors influencing the spread of equine influenza onto horse premises [30], perceptions of outbreak management [31] and factors associated with the perception of on-farm biosecurity effectiveness [32]. Ultimately, 200 managers were enrolled, of which 127 had at least one confirmed case of equine influenza at their premises.

Face-to-face interviews were conducted by two trained interviewers (KS and SMF) between July and October 2009, based on a structured questionnaire. The two interviewers piloted the questionnaire on four horse managers to test questions for ambiguity and to ensure similar method and response recording. Of the 61 questions included in the interview, 15 closed or semi-closed questions were used in the present analysis. Questions were designed to obtain information about the demographics of managers, the nature of their involvement with horses, their sources of information about infection control during the 2007 equine influenza outbreak, their horse’s equine influenza infection status during the 2007 outbreak and their attitudes towards a potential future outbreak of equine influenza (perceived likelihood and preparedness).

A purpose-built Microsoft Access 2007 database (Microsoft Corporation, Redmond, WA, USA) was created for data entry. Data cleaning and statistical analyses were conducted using SAS statistical software (release 9.2 © 2002–2008, SAS Institute Inc., Cary, NC, USA).

Ethical approval for the study was obtained from the University of Sydney’s Human Research Ethics Committee (07-2009/11840).

### Outcome and explanatory variables

The outcome variable was based on a question asking horse managers to rate their level of vulnerability to a future equine influenza outbreak on a four-point scale: very vulnerable, vulnerable, not vulnerable, or unsure. Observations from participants selecting the unsure category of this question were omitted. The resulting three-category outcome variable was used in ordinal logistic regression analyses to investigate the association of explanatory variables with horse managers’ perceived vulnerability. The 32 explanatory variables investigated in this study are related to manager and premises description, sources of infection control information used during the outbreak, outbreak experience and perceptions concerning a potential future equine influenza outbreak (Table 1).

**Table 1:** Explanatory variables analysed for associations with the perception of perceived vulnerability to a future equine influenza outbreak based on a study of 200 horse owners and managers, conducted in 2009 in NSW, Australia

**Variable group**	**Variables**
Manager demographics and premises descriptors	Age^a^; Gender^b^; Number of horses^c^; Involved in horse competition/sporting events^d^; Involved in horse racing; Involved in equestrian events^e^; Involved in rodeo-style horse events^f^; Horse breeders; Horse agistment or spelling facility^g^; Horses kept only for recreation; Horses used for farm work. Premises enterprise type^b^; Income derived from horses^a^; Regional cluster^b^.
Sources of infection control information during the outbreak	Internet; General media (television, newspaper, radio); Other horse owners; Veterinarians; Other horse professionals (farriers, dentists, chiropractors, trainers, coaches); Australian Horse Industry Council; State Department of Primary Industries (NSW DPI); Association/society (breed, sporting^d^); Horse equipment or feed retailer.
Equine influenza outbreak experience and perceptions	Premises infected during the 2007 outbreak; Suspected transmission route^b^; Outbreak control zone^b^; Perceived stringency of own outbreak biosecurity measures^a^; Perceived level of own outbreak stringency compared to horse owners in the neighbourhood^a^; Perceived level of preparedness for a future equine influenza outbreak^a^. General interest in infection control information^a^; Perceived likelihood of a future equine influenza outbreak in Australia in the next five years^a^; Perceived likelihood of a future other exotic equine disease outbreak in Australia in the next five years^a^.

All variables are binary (1 = Yes, 0 = No) unless indicated otherwise. ^a^Ordinal variable; ^b^Categorical variable; ^c^Continuous variable; ^d^Competition/sporting events include horse showing, pony-club, rodeo-style, polo, equestrian and/or racing; ^e^Equestrian events include dressage, showjumping, eventing, and endurance. ^f^Rodeo-style horse events include camp-drafting, cutting and barrel-racing; ^g^Agistment is the keeping of other owners’ horses for remuneration, whilst spelling is resting horses on pasture.

### Data analyses

To assess the distribution of explanatory variables, contingency tables for categorical variables and summary statistics for continuous variables (alone and classified by the outcome) were created. Unconditional associations between each of the explanatory variables and the outcome variable (‘perceived vulnerability’) were assessed using univariable ordinal logistic regression, with UniLogistic SAS macro [33]. Explanatory variables associated with the outcome variable at *p* < 0.20 (based on the likelihood-ratio chi-square test) were checked for missing values and tested for pair-wise collinearity by calculating Spearman’s rank correlation coefficient (*ρ*) for pairs of ordinal variables or by performing Pearson chi-square test for other pairs of variables. Variables were considered collinear if *ρ* > 0.7 or *p* < 0.05. The assumption of linearity for the single continuous variable (‘Number of horses’) was assessed by categorising it into quartiles and plotting mid-points of these quartiles against their respective regression coefficients [34].

A manual forward stepwise approach was then used for model building. Multivariable ordinal logistic regression models were built using MultiLogistic SAS macro (available at http://sydney.edu.au/vetscience/biostat/macros/index.shtml). Only variables associated with the outcome at a *p*-value <0.05 were retained in the final model. All variables eligible for multivariable analyses were retested by adding them one-at-a-time to the final model and measuring for a >20% change in any regression coefficient. All analyses were adjusted for the gender and age of the managers, as these two variables were considered as *a priori* confounders based on other research that has shown they can be key determinants of perceptions about health protective behaviour [35,36].

All biologically meaningful two-way interactions amongst variables in the final model were tested for statistical significance at *p* < 0.05. Potential interviewer bias was assessed by addition of an interviewer-level random effect term to the final model [37], enabling calculation of the intra-class correlation (ICC) to assess for clustering of observations by interviewer [38]. The proportional odds assumption of the cumulative logit model was tested by the score test [39]. Model fit was assessed using the Deviance goodness-of-fit chi-square statistic.

## Results

The interviewed managers were predominantly female (68%) and middle-aged (35–54 years old, 74%). Almost half of the interviewees managed horses on small acreage premises (47%), 18% held horses at a horse or rider training facility, another 18% had horses on a farm with commercial livestock or cropping enterprises, 9% were from a commercial horse breeding enterprise and 8% managed agistment premises where horses of multiple owners are kept together, typically for remuneration.

Of all interviewees, 60 (31.4%) perceived themselves to be ‘not vulnerable’, 70 (36.6%) perceived themselves to be ‘vulnerable’ and 61 (31.9%) perceived themselves to be ‘very vulnerable’.

Contingency tables for 17 explanatory variables associated (*p* ≤ 0.20) with ‘perceived vulnerability’ in univariable analyses are presented in Table 2. Of these, one pair of variables (‘Premises infected during the 2007 outbreak’ and ‘Suspected equine influenza transmission route during the 2007 outbreak’) were highly collinear. The one of the two variables less strongly associated with the outcome (‘Premise infected during the 2007 outbreak’) was therefore excluded from all further analyses. No variable had >10% missing observations. Multivariate model building using the remaining 16 variables resulted in a final model that included eight variables (Table 3). Managers involved in horse racing and those with horse premises located in rural areas further away from Sydney, in the Hunter Valley or the Northern NSW geographic regions, had decreased levels of perceived vulnerability to a future incursion (Table 3).

**Table 2:** **Contingency tables and univariable ordinal logistic regression results for the association of explanatory variables with the perception of being not vulnerable to a future equine influenza outbreak (*p*** **< 0.20) based on responses of 191 horse owners and managers interviewed in 2009 in NSW, Australia**

		**Perception of vulnerability**				
		***Not vulnerable***	***Vulnerable***	***Very vulnerable***	**Odds-ratios**^**a **^**(95% CI)**	
		**Freq**	**Freq**	**Freq**		
**Variables and categories**		**(Row%)**	**(Row%)**	**(Row%)**		***P***
Regional cluster						<0.001
	Northern NSW	23 (50%)	18 (39%)	5 (11%)	8.32 (3.78, 18.89)	
	South-West Sydney	9 (30%)	13 (43%)	8 (27%)	3.52 (1.50, 8.44)	
	Hunter Valley	13 (30%)	18 (42%)	12 (28%)	3.44 (1.58, 7.63)	
	Central Coast	4 (20%)	12 (60%)	4 (20%)	3.25 (1.27, 8.45)	
	North-West Sydney	11 (20%)	10 (18%)	34 (62%)	1	
Equine influenza outbreak control zone						<0.001
	Restricted zone	45 (34%)	60 (46%)	26 (20%)	3.66 (2.01, 6.79)	
	Special restricted zone	15 (24%)	11 (17%)	37 (59%)	1	
Suspected equine influenza transmission route during the 2007 outbreak						<0.001
	Wind	16 (21%)	24 (31%)	37 (48%)	0.27 (0.14, 0.50)	
	Direct/indirect	14 (30%)	18 (38%)	15 (32%)	0.51 (0.25, 1.01)	
	Not infected	30 (43%)	29 (41%)	11 (16%)	1	
Premises infected during the 2007 outbreak						<0.001
	Yes	30 (24%)	42 (34%)	52 (42%)	0.35 (0.20, 0.60)	
	No	30 (43%)	29 (41%)	11 (16%)	1	
Premise enterprise type						0.001
	Farm	18 (51%)	14 (40%)	3 (9%)	4.02 (1.93, 8.59)	
	Stud	8 (47%)	5 (29%)	4 (24%)	2.69 (1.01, 7.45)	
	Agistment^b^	6 (38%)	4 (25%)	6 (38%)	1.48 (0.53, 4.18)	
	Training	7 (20%)	14 (40%)	14 (40%)	0.93 (0.45, 1.90)	
	Small acreage home	21 (23%)	34 (37%)	36 (40%)	1	
Involved in equestrian events^c^						0.003
	Yes	13 (21%)	19 (31%)	29 (38%)	0.42 (0.23, 0.74)	
	No	47 (35%)	52 (39%)	34 (26%)	1	
Involved in rodeo-style horse events^d^						0.006
	Yes	14 (45%)	14 (45%)	3 (10%)	2.68 (1.33, 5.51)	
	No	46 (28%)	57 (35%)	60 (37%)	1	
Involved in horse racing						0.193
	Yes	8 (42%)	7 (37%)	4 (21%)	1.78 (0.75, 4.37)	
	No	52 (30%)	64 (36%)	59 (34%)	1	
Received infection control information from sporting/breeding association/society^e^						0.003
	Yes	23 (21%)	44 (41%)	41 (38%)	0.45 (0.26, 0.76)	
	No	37 (43%)	27 (31%)	22 (26%)	1	
Received infection control information from non-veterinarian horse professionals^f^						0.004
	Yes	11 (18%)	24 (39%)	27 (44%)	0.44 (0.25, 0.76)	
	No	49 (37%)	47 (36%)	36 (27%)	1	
Received infection control information from the internet						0.034
	Yes	43 (28%)	55 (36%)	55 (36%)	0.54 (0.26, 0.95)	
	No	17 (41%)	16 (39%)	8 (20%)	1	
Received infection control information from other horse owners						0.119
	Yes	40 (29%)	48 (35%)	50 (36%)	0.64 (0.36, 1.12)	
	No	20 (36%)	23 (41%)	13 (23%)	1	
Perceived stringency of own outbreak biosecurity measures						0.039
	Very stringent/stringent	34 (26%)	49 (37%)	49 (37%)	0.51 (0.28, 0.92)	
	Average/normal	21 (41%)	18 (35%)	12 (24%)	1	
	Less/not at all stringent	5 (46%)	4 (36%)	2 (18%)	1.25 (0.37, 4.36)	
Perceived level of preparedness for a future equine influenza outbreak						0.042
	Unprepared	4 (17%)	7 (29%)	13 (54%)	1	
	Prepared	35 (32%)	45 (42%)	28 (26%)	2.92 (1.26, 7.03)	
	Highly prepared	21 (36%)	18 (30%)	20 (34%)	2.63 (1.06, 6.73)	
Perceived level of general interest in infection control						0.066
	Very interested	28 (27%)	38 (36%)	39 (37%)	0.48 (0.19, 1.18)	
	Interested	20 (30%)	27 (41%)	19 (29%)	0.36 (0.15, 0.86)	
	Not interested	12 (52%)	6 (26%)	5 (22%)	1	
Age (years)						0.045
	>54	12 (40%)	11 (37%)	7 (23%)	0.75 (0.26, 2.11)	
	35-54	37 (26%)	54 (38%)	51 (36%)	0.40 (0.17, 0.95)	
	<35	11 (50%)	6 (27%)	5 (23%)	1	
Gender						0.126
	Male	22 (35%)	26 (41%)	15 (24%)	1.54 (0.89, 2.68)	
	Female	38 (29%)	45 (34%)	48 (37%)	1	

^a^Odds ratios are based on cumulative logit model and compare the odds of being not vulnerable versus being vulnerable or very vulnerable. For example, the odds ratio of 3.66 for the variable ‘Equine influenza control zone during the 2007 outbreak’ indicates that owners/managers residing in the restricted area in 2007 are 3.66 times more likely to perceive that they are not vulnerable to a future outbreak than those who resided in the special restricted area; ^b^Agistment is the keeping of other owner’s horses for remuneration, whilst spelling is resting horses on pasture; ^c^Equestrian events include dressage, showjumping, eventing, and endurance; ^d^Rodeo-style horse events include camp-drafting, cutting and barrel-racing; ^e^Sporting organisations include those relevant to horse showing, pony-club, rodeo-style, polo, equestrian and/or racing; ^f^including farriers, dentists, chiropractors, trainers and coaches.

**Table 3:** Final ordinal logistic regression model of 191 horse owners and managers perception of being not vulnerable to a future equine influenza outbreak, based on a study conducted in New South Wales, Australia in 2009

**Parameters**		**b**	**SE(b)**	**Adjusted odds ratio**	**(95% CI)**	***P*-value**^**a**^
Constant 1		−1.12	0.72	-	-	-
Constant 2		0.88	0.72	-	-	-
Received infection control information from a sporting/breeding association/society^b^		-	-	-	-	<0.001
	No	0	-	1	-	-
	Yes	−1.11	0.31	0.33	(0.18, 0.61)	-
Received infection control information from a non-veterinarian horse professional^c^		-	-	-	-	0.04
	No	0	-	1	-	-
	Yes	−0.65	0.33	0.52	(0.27, 0.99)	-
Involved in horse racing		-	-	-	-	0.006
	No	0	-	1	-	-
	Yes	1.54	0.55	4.67	(1.63, 13.93)	-
Geographic cluster		-	-	-	-	0.024
	NW Sydney	0	-	1	-	-
	Central Coast	1.01	0.56	2.74	(0.96, 7.94)	
	Hunter Valley	1.09	0.45	2.97	(1.22, 7.38)	
	Northern NSW	1.67	0.51	5.30	(1.96, 14.78)	
	SW Sydney	0.78	0.48	2.19	(0.84, 5.76)	
Suspected equine influenza transmission route during the 2007 outbreak		-	-	-	-	0.035
	Not infected	0	-	1	-	-
	Direct or indirect transmission	−0.57	0.40	0.56	(0.26, 1.24)	-
	Wind transmission	−0.98	0.38	0.37	(0.18, 0.78)	-
Perceived preparedness for a future equine influenza outbreak		-	-	-	-	0.023
	Unprepared	0	-	1	-	-
	Prepared	0.99	0.51	2.68	(1.00, 7.53)	-
	Highly prepared	1.47	0.54	4.34	(1.52, 13.11)	-
Age		-	-	-	-	0.323
	<35 years	0	-	1	-	-
	35-54 years	−0.68	0.47	0.51	(0.19, 1.29)	-
	≥55 years	−0.44	0.59	0.65	(0.20, 2.10)	-
Gender		-	-	-	-	0.864
	Female	0	-	1	-	-
	Male	−0.06	0.34	0.94	(0.48, 1.83)	-

Log likelihood = 64.81; d.f. = 14; *P* < 0.001; Goodness-of-fit deviance χ^2^-test statistic *P*-value = 0.52; Score test = 0.32.

^a^*P*-values based on Wald χ^2^-test of significance; ^b^ Sporting organisations include those relevant to horse showing, pony-club, rodeo-style, polo, equestrian and/or racing; ^c^ including farriers, dentists, chiropractors, trainers and coaches.

Managers of premises infected during the 2007 outbreak (measured as suspected transmission via direct/indirect contact or via wind versus not infected), had increased levels of perceived vulnerability to a future outbreak. Similarly, increased levels of perceived vulnerability were also found among managers, who received infection control information during the 2007 equine influenza outbreak from either a sporting or breeding association or society or from a non-veterinarian horse professional such as a farrier, chiropractor, dentist, horse trainer or riding coach.

None of the two-way interaction terms between variables in the final model were significantly associated with the outcome variable. Intra-class correlation for clustering of responses according to interviewer was effectively zero. The score test for the proportional odds assumption was non-significant (p = 0.32) indicating that the cumulative logit model was appropriate for the final model [40].

## Discussion

This study provides objective evidence that perceptions of vulnerability to a future equine influenza outbreak differ among different horse industry participants. Many authors [20-22] have argued that risk perception needs to be understood in order to design effective animal health biosecurity programs. Protection motivation theory suggests that a heightened threat appraisal (supported by high levels of perceived severity and vulnerability and inhibited by intrinsic and extrinsic rewards for non-compliance) generally elicits positive behavioural intention and compliance in the presence of a sufficient coping appraisal, yet a lack of perceived vulnerability is unfavourable to the motivation to perform a protective behaviour [27,28]. Hence, in this study, factors associated with a lack of perceived vulnerability were examined, as these were considered most likely to link to low levels of biosecurity practice.

Three factors were associated with reduced levels of perceived vulnerability to a future outbreak in multivariable analyses, namely involvement in horse racing, geographical location during the 2007 equine influenza outbreak and perceived high preparedness for a future equine influenza outbreak. Those managers involved in horse racing had reduced levels of perceived vulnerability compared to those who were not involved in this industry sector. Previously the Australian horse racing industry has demanded ongoing vaccination for equine influenza as the disease is widespread worldwide and all other significant thoroughbred racing nations vaccinate horses routinely [41]. Lower perceived vulnerability in this sector could be explained by the fact that the racing industry was better supported by the response agencies during the 2007 outbreak when compared to other sectors [41,42] and by lower risk perceptions regarding potential impacts for international horse trade and travel for overseas race meetings.

Premises location in more rural areas may explain reduced levels of perceived vulnerability associated with certain geographical clusters as found in this study. Managers from rural areas may feel less at risk due to being less connected with other horses; however, early local disease spread occurred predominantly in rural areas during the 2007 outbreak [15]. The areas in and surrounding Sydney (Central Coast area) characterised by increased levels of perceived vulnerability in this study, have greater concentrations of horses and they experienced higher incidence of equine influenza during the 2007 outbreak [14,15], so increased vulnerability.

In addition to involvement with horse racing and geographical location, perceived preparedness to a future outbreak was also associated with perceived vulnerability: managers who reported being highly prepared reported reduced levels of perceived vulnerability. Contrary to the findings of this study, previous work hypothesised that a heightened sense of preparedness and control may be linked to increased vulnerability as it found that perceived high preparedness to a future equine influenza outbreak was associated with perceived high effectiveness of biosecurity measures [32]. The reduced perceived vulnerability associated with perceived high preparedness found in the current study may be logically explained: managers may have a heightened sense of control and self-efficacy and feel more prepared due to their reduced risk perceptions and knowledge on what to do regarding a future outbreak, based on their previous experience in 2007.

In addition to factors associated with reduced levels of perceived vulnerability to a future equine influenza outbreak, this study also identified factors associated with increased levels of perceived vulnerability: having previously encountered equine influenza infection and the seeking of infection control information from various sources.

Multivariable analyses results indicate that managers whose horses were infected with equine influenza perceived increased levels of vulnerability to a future outbreak of equine influenza compared to those managing uninfected horses during 2007. Furthermore, managers who thought their horses were infected via wind transmission during the 2007 outbreak reported increased levels of vulnerability to a future outbreak compared to those who believed their horses had been infected by other means of transmission. A similar association was found for perceived effectiveness of biosecurity measures and perceived source of transmission [32], and is probably explained by a combination of managers’ realisations of the highly contagious nature of the disease and a lack of perceived personal control over the spread of infection.

The utilisation of two specific sources of infection control information used during the 2007 outbreak was significantly associated with increased levels of perceived vulnerability to a future outbreak, namely sporting or breeding associations/societies and non-veterinarian horse professionals. The findings that the two information sources are significantly associated with the outcome can be explained in two ways: these sources are providing information that is either accurate or inaccurate. In the case of the 2007 equine influenza outbreak, there is evidence that these sources were accurate. If the information provided was inaccurate, then horse managers may have been vulnerable due to having been misinformed by these sources, similar to another study examining pandemic flu vaccination uptake [36]. However, in 2007 the Australian structure for emergency animal disease management provided good overall government-industry interaction and communications [43]. Consequently, it can be assumed that information provided by industry organisations to their members was based on information supplied by government veterinarians and hence accurate. So, if associations/societies or non-veterinarian horse professionals, were providing accurate information, then the study findings may imply that managers, who received information from them were more aware and concerned about the disease threat, and hence more engaged. However, the associations with non-veterinarian horse professionals and industry organisations information sources may also be a proxy measure for having experienced more impacts associated with control measures during 2007, such as the cancellation of events and inability to breed horses due to movement restrictions [12,17,19]. Another aspect that could be contributing here is: trusted sources are regarded as the best provider of infection control information [44]. ‘Sporting or breeding associations/societies’ and ‘non-veterinarian horse professionals’ represent information sources which are based on professional contact networks. Their inclusion in the final model may reflect managers established relationships with them and trust in their professionalism and in the accuracy of the supplied information [44].

### Study strengths and limitations

Observational studies are subject to a number of sources of bias. In this study, selection bias is likely due to selection from the NSW DPI laboratory databases for the concurrently conducted case–control study [30]. This limits the external validity and generalisability of the results, yet no other sampling frame for Australian horse owners was available due to a lack of legal requirements for registration of horses in Australia. However, the age and gender distributions of the study sample, representing greater proportions of middle-aged and female horse owners, is similar to that in a previous study conducted by the Australian Horse Industry Council among its members [45], suggesting that the sample is representative of the Australian horse industry. Studies concerning human pandemic influenza found that older people and males had higher intentions and uptake of vaccination [36], however, more relevant to this study, studies of protective hygiene and quarantine behaviours during human pandemics found that, if there are gender differences, woman are consistently more likely to carry out the behaviour [35,46]. The evidence for age is less conclusive but the balance of evidence indicates that older people are more likely to carry out protective behaviours [35]. The high proportion of middle-aged woman in this study may result in greater levels of threat appraisal and motivation in this sample compared to the general population of horse managers, but both age and gender were adjusted for in multivariable models in this study. Hence, due to recognition *a priori* of the influence of gender and age, the associations discussed are based on adjusted measures found significant after accounting for gender and age and other variables in the final model.

To achieve better cooperation and rapport and more consistent, reliable and complete data, face-to-face on-farm interviews were conducted in this study. Open discussions led up to the questioning to stimulate memory of the 2007 outbreak, however, some degree of recall bias is possible.

## Conclusions

This study identified differing levels of perceived vulnerability to a future equine influenza outbreak among horse managers, likely explained by different levels of financial and sporting involvement with horses, geographical location and prior experience with equine influenza during the 2007 epidemic. To achieve greater horse manager compliance with biosecurity programs it is recommended that further engagement with managers, particularly in the horse racing sector and rural areas, be conducted. A greater level of engagement should be accomplished by delivery of information on risks together with appropriate response measures via trusted sources, to increase horse managers’ sense of control. This will ensure that a lack of perceived vulnerability does not result in complacency or a lack of preparedness for a future outbreak.

## Competing interest

The authors declare that they have no competing interests.

## Authors’ contributions

KS was involved in all aspects of the study; design, data collection, data analysis, drafting and editing the paper. SF assisted with the design of the study, collected data and provided input in editing of the paper. MT, JAT, MW and ND assisted with the study design and provided input in all aspects of the review, data interpretation and editing of the paper. All authors read and approved the final manuscript.
